# Understanding Autoimmune Mechanisms in Multiple Sclerosis Using Gene Expression Microarrays: Treatment Effect and Cytokine-related Pathways

**DOI:** 10.1080/17402520400001603

**Published:** 2004

**Authors:** A. Achiron, M. Gurevich, D. Magalashvili, I. Kishner, M. Dolev, M. Mandel

**Affiliations:** 1 Multiple Sclerosis Center and Neurogenomics Unit Sheba Medical Center and Sackler School of Medicine Tel-Aviv University hashomer Israel; 2 Neurogenomics Unit Sheba Medical Center and Sackler School of Medicine Tel-Aviv University hashomer Israel; 3 Blood Bank Sheba Medical Center and Sackler School of Medicine Tel-Aviv University hashomer Israel

## Abstract

Multiple sclerosis (MS) is a central nervous system disease in which activated autoreactive T-cells invade the blood brain barrier and initiate an inflammatory response that leads to myelin destruction and axonal loss. The etiology of MS, as well as the mechanisms associated with its unexpected onset, the unpredictable clinical course spanning decades, and the different rates of progression leading to disability over time, remains an enigma. We have applied gene expression microarrays technology in peripheral blood mononuclear cells (PBMC) to better understand MS pathogenesis and better target treatment approaches. A signature of 535 genes were found to distinguish immunomodulatory treatment effects between 13 treated and 13 untreated MS patients. In addition, the expression pattern of 1109 gene transcripts that were previously reported to significantly differentiate between MS patients and healthy subjects were further analyzed to study the effect of cytokine-related pathways on disease pathogenesis. When relative gene expression for 26 MS patients was compared to 18 healthy controls, 30 genes related to various cytokine-associated pathways were identified. These genes belong to a variety of families such as interleukins, small inducible cytokine subfamily and tumor necrosis factor ligand and receptor. Further analysis disclosed seven cytokine-associated genes within the immunomodulatory treatment signature, and two cytokine-associated genes SCYA4 (small inducible cytokine A4) and FCAR (Fc fragment of IgA, CD89) that were common to both the MS gene expression signature and the immunomodulatory treatment gene expression signature. Our results indicate that cytokine-associated genes are involved in various pathogenic pathways in MS and also related to immunomodulatory treatment effects.


*Clinical & Developmental Immunology*, September/December 2004, Vol. 11 (3/4), pp. 299–305

Taylor & Francis healthsciences

### Understanding Autoimmune Mechanisms in Multiple Sclerosis Using Gene Expression Microarrays: Treatment Effect and Cytokine-related Pathways

A. ACHIRON[a,b,*,†], M. GUREVICH[b,†], D. MAGALASHVILI[a], I. KISHNER[a], M. DOLEV[a] and M. MANDEL[c]

[a]*Multiple Sclerosis Center and Neurogenomics Unit, Sheba Medical Center, Tel-Hashomer, and Sackler School of Medicine, Tel-Aviv University, 52621, Israel;* [b]*Neurogenomics Unit, Sheba Medical Center, Tel-Hashomer, and Sackler School of Medicine, Tel-Aviv University, 52621, Israel;* [c]*Blood Bank, Sheba Medical Center, Tel-Hashomer, and Sackler School of Medicine, Tel-Aviv University, 52621, Israel*

Multiple sclerosis (MS) is a central nervous system disease in which activated autoreactive T-cells invade the blood brain barrier and initiate an inflammatory response that leads to myelin destruction and axonal loss. The etiology of MS, as well as the mechanisms associated with its unexpected onset, the unpredictable clinical course spanning decades, and the different rates of progression leading to disability over time, remains an enigma. We have applied gene expression microarrays technology in peripheral blood mononuclear cells (PBMC) to better understand MS pathogenesis and better target treatment approaches. A signature of 535 genes were found to distinguish immunomodulatory treatment effects between 13 treated and 13 untreated MS patients. In addition, the expression pattern of 1109 gene transcripts that were previously reported to significantly differentiate between MS patients and healthy subjects were further analyzed to study the effect of cytokine-related pathways on disease pathogenesis. When relative gene expression for 26 MS patients was compared to 18 healthy controls, 30 genes related to various cytokine-associated pathways were identified. These genes belong to a variety of families such as interleukins, small inducible cytokine subfamily and tumor necrosis factor ligand and receptor. Further analysis disclosed seven cytokine-associated genes within the immunomodulatory treatment signature, and two cytokine-associated genes SCYA4 (small inducible cytokine A4) and FCAR (Fc fragment of IgA, CD89) that were common to both the MS gene expression signature and the immunomodulatory treatment gene expression signature. Our results indicate that cytokine-associated genes are involved in various pathogenic pathways in MS and also related to immunomodulatory treatment effects.

*Keywords*: Multiple sclerosis; Gene expression; Cytokine; Immunomodulatory treatment

### INTRODUCTION

Multiple sclerosis (MS) is a central nervous system disease with an unpredictable clinical course and outcome. A variety of genetic, immunologic and environmental factors have been implicated in triggering the onset and progression of the disease. Genetic background may play a role in disease pathogenesis as MS is more common in Caucasians and disease frequency increases with distance from the Equator in both hemispheres (Kenealy *et al.*, 2003). Pathologically, the disease is characterized by perivascular infiltration of monocytes and lymphocytes mainly CD4 cells within the brain and spinal cord that lead to myelin destruction (Prat and Martin, 2002). Peripheral blood mononuclear cells (PBMC) are involved in the pathogenesis of the disease and induce active demyelination. Autoreactive activated T-cells invade the blood brain barrier and initiate an inflammatory response that leads to myelin destruction and significant neurological disability. However, the etiology of MS, as well as the mechanisms associated with its unexpected onset, the unpredictable clinical course spanning decades, and the different rates of progression leading to disability over time, all still remain enigmas. New approaches are needed to better understand MS pathogenesis and in order to better target treatment approaches to identify patients with poor prognosis. Gene expression microarray technology is a new tool for comprehensively detecting and quantifying tens and thousands of gene transcripts simultaneously (Kolbert *et al.*, 2003). This parallel quantification of large number of messenger RNA transcripts provides detailed insight into cellular processes

*Corresponding author. Tel.: +972-3-5303811. Fax: +972-3-5348186. E-mail: achiron@post.tau.ac.il
†Contributed equally to this paper.

ISSN 1740-2522 print/ISSN 1740-2530 online © 2004 Taylor & Francis Ltd
DOI: 10.1080/17402520400001603

involved in the regulation of gene expression, and allows new understanding of signaling networks that operate within cells or tissues and of the molecular processes involved (Watson *et al.*, 1998). Specifically in MS, which is considered a multi-factorial and heterogeneous disease, research interest is not aimed at finding a single change in gene expression that might be the key to different disease phenotypes, but rather at evaluating overall patterns of gene expression in order to understand the architecture of genetic regulatory networks involved in the disease (Baranzini and Hauser, 2002). Thus, instead of looking for a needle in a haystack, microarrays technology allows a global approach that could ultimately lead to identification of the transcription-control mechanisms operating the pathological disease processes.

In a recent study we have reported the identification of a statistically significant transcriptional signature of 1109 genes expressed in PBMC from 26 MS patients using oligonucleotide microarrays (Achiron *et al.*, 2004). The MS signature contained genes involved in T-cell activation and expansion, inflammation and apoptosis, and was irrespective to the disease activation state or immunomodulatory treatment. Another transcriptional signature consisting of 721 genes involved in cellular recruitment, epitope spreading, and escape from regulatory immune surveillance, identified MS patients in acute relapse compared to remission.

In the current study, we studied the effect of immunomodulatory treatment on MS gene expression signature, and further evaluated the autoimmune mechanisms involved by specifically assessing cytokine-associated genes expression.

Cytokines have crucial functions in the development, differentiation and regulation of immune cells. As a result, dysregulation of cytokine production or action is thought to have a central role in the development of autoimmunity and autoimmune diseases such as MS. Moreover, the fact that the new immunomodulatory drugs (i.e. beta-interferons, glatiramer acetate and intravenous imunoglobulins) used for the treatment of MS, are though to affect and modulate the autoreactive immune response through the cytokine pathways (Dhib-Jalbut, 2002), prompt us to investigate whether specific expression of gene transcripts is associated with alteration of cytokine levels and immunomodulatory treatment effects.

### METHODS

### Patients

Twenty-six patients with definite MS and a relapsing-remitting disease course were included in the PBMC gene expression study and participated in the cytokine expression and immunomodulatory treatment effect analyses. The clinical and demographic variables of the study patients were previously reported (Achiron *et al.*, 2004). Thirteen patients were on immunomodulatory treatments (interferon beta-1a, interferon beta-1b, glatiramer acetate and IVIg) for at least 3 months prior to gene expression study, and 13 patients were naïve to immunomodulatory treatment. All patients had peripheral blood counts within the normal range. The Sheba Medical Center Institutional Review Board approved the study, and all patients gave written informed consent for participation.

### RNA Isolation and Microarray Expression Profiling

PBMC were separated on ficol hypaque gradient, total RNA was purified, labeled, hybridized to a 12,000 Genechip array (U95Av2) and scanned (Hewlett Packard, GeneArray-TM scanner G2500A) according to the manufacturer's protocol (Affymetrix Inc, Santa Clara, CA). MAS5 software (Affymetrix Inc.) was used to analyze the scanned arrays. All data were normalized by dChip software (Li and Wong, 2001). Probes that did not have an expression value of 100 in at least one of the arrays were filtered. The hybridization of the arrays was done in eight batches. A Unigene cluster was assigned to each of the Affymetrix probe sets to obtain gene specific annotation.

### Statistical Analysis

Statistical analysis was performed using the ScoreGenes software tools (http://compbio.cs.huji.ac.il/scoregenes/). The gene expression profile of each patient was normalized to the median gene expression profile for the entire sample as previously described (Achiron *et al.*, 2004). The data were analyzed by the classic parametric *t*-test, and the non-parametric tests the threshold number of misclassifications (TNoM) method and the Info score to identify differences in mean gene expression levels between comparison groups (Kaminski and Friedman, 2002). Fold change was calculated for each gene in the samples against the geometric mean of controls and log (base 2) transformed. The most informative differentially expressed genes were defined as those that pass 95% confidence interval on all three statistical tests (*t*-test, TNoM and Info).

Evaluation of immunomodulatory treatment effect by gene expression to assess specific gene transcripts associated with treatment was performed using a 12,000 Genechip array. The 1109 most informative genes that differentiated MS patients from healthy subjects (Achiron *et al.*, 2004), served to investigate a specific autoimmune signature associated with cytokine-related gene expression. To control for artifacts of batches we fitted a multiple effect model for each gene, where, we modeled the log-ratio measurement as a sum of contributions of (a) batch, (b) subject state (control, MS), (c) treatment and (d) array specific noise. We fitted the model to minimize the least sum of squares of the errors, and created a cleaned log-ratio file by removing from each log-ratio the associated batch effect parameter. Spotfire DecisionCite for Functional Genomics software was used for treatment effect analyses.

### RESULTS

### Gene Expression Patterns Identify Immunomodulatory Treatment Effects in MS

Gene expression patterns between treated and untreated MS patients differed by a set of 535 genes (represented by 539 probes). Although patients were on various immunomodulatory treatments for different periods of time, these 535 genes were differentially expressed ($p < 0.05$ in all three statistical scoring tests) between treated and untreated MS patients. Of these, only 57 were among the 1109 genes within the MS expression signature, and 34 were among the 721 genes related to the disease phase transcriptional signature. Three-dimensional Scatter Plot of these genes (Fig. 1A) clearly demonstrates a difference between treated and untreated MS patients. An example of significantly differentiating genes with a differential behavioral pattern between treated and untreated patients is shown in relapse (Fig. 1B) and in remission (Fig. 1C).

### Cytokine-related Gene Transcripts in MS

Analysis of the expression pattern of the 1109 gene transcripts that significantly differentiate between MS patients and healthy subjects to study the cytokine-related gene transcripts demonstrated 30 genes that passed 95% FDR and exhibited ($p < 0.05$) in all three statistical scores, (Table I). Most of these genes belong to a variety of cytokine families such as TNF ligand and its receptor, interleukins and small inducible cytokine subfamily. There was no effect of immunomodulatory treatments on the cytokine-related genes within the MS signature, as analysis of the non-treated MS patients resulted in the same level of expression of these genes.

### Cytokine-related Gene Transcripts Associated with Immunomodulatory treatment in MS

Further analysis of the 535 treatment-related genes disclosed seven cytokine-related genes within the MS immunomodulatory treatment signature, (Table II). Two genes small inducible cytokine A4 (SCYA4) and Fc fragment of IgA, CD89 (FCAR) were common to the cytokine-related pathways within both the MS signature and the immunomodulatory treatment signature.

### DISCUSSION

In the current study our findings demonstrate that immunomodulatory treatment transcriptional signature can be identified by microarray analysis. As the MS patients included in the study were treated with various immunomodulatory treatments and also differed in relation to treatment duration, the possibility to define specific drug effects was limited. Even though, we identified specific treatment related gene pattern that differentiated between treated and untreated patients. Although currently the precise mechanisms of action of immunomodulatory treatments in MS are not fully understood, it is conceivable that in the future, assessment of specific pathways related to treatment effects could be analyzed by gene microarray technology. Identification of these pathways will serve to predict patients that are responders or non-responders to immunomodulatory interventions even before treatment is initiated.

The second question evaluated in the current study was related to the role of cytokine pathways in MS. Numerous studies have addressed this question, often with conflicting results; elevated, normal and decreased levels of almost all cytokines have been reported (Martino *et al.*, 2000; Ozenci *et al.*, 2002). In the current study we demonstrated within the MS specific gene expression signature, several distinguished groups of cytokine related genes responsible for migration of inflammatory cells and T-cell mediated immune response regulation. These include the chemokine group ligands CXCL, interleukins and their receptors, TNF family and small inducible cytokine family. These genes participate in various pathogenic pathways involved in MS including inflammatory cell migration (CXCL1, CXCL2, CXCL3, IL8, SCYA2, SCYA4, SCYA20, SCYE1), T-cell activation and expansion (NFATC3, IL15, IL2RB), apoptosis (TNFRSF4, TNF, TNFSF6, MAP2K3), demyelination (SCYA20, IL1b, IL1R1) and immune regulation (IL6, CEBPB). We therefore, suggest the following sequence of cytokine-related events to play a role in MS pathogenesis (Fig. 2).

### Inflammatory Cell Migration

MS is considered as a T-cell-mediated inflammatory demyelinating disease in which the immune system is tricked to first see central nervous system myelin as foreign and then to destroy it (Trapp, 2004). Although the trigger that induces T-cell-mediated myelin destruction has not yet been identified, we suggest that once the autoimmune process has been initiated it involves inflammatory cell migration into the central nervous system. The chemokine group ligands CXCL1 (GRO1), CXCL2 (GRO2) and CXCL3 (GRO3) are ligands to the CXC chemokine subfamily. CXCL1 is a mitogenic factor involved in inflammatory processes, with a chemotactic activity for neutrophils, and is known to regulate embryonic oligodendrocyte precursor migration (Tsai *et al.*, 2002). CXCL2 is produced by activated monocytes and neutrophils, expressed at sites of inflammation and serves as a chemotactic agent for polymorphonuclear leucocytes (Wolpe *et al.*, 1989). CXCL3 is a potent neutrophil chemoattractant both *in vitro* and *in vivo*, is up regulated simultaneously with symptom onset of acute experimental autoimmune encephalomyelitis, the animal model of MS, and its expression correlates with the intensity of inflammation in the central nervous system (Glabinski *et al.*, 1998). IL-8 is

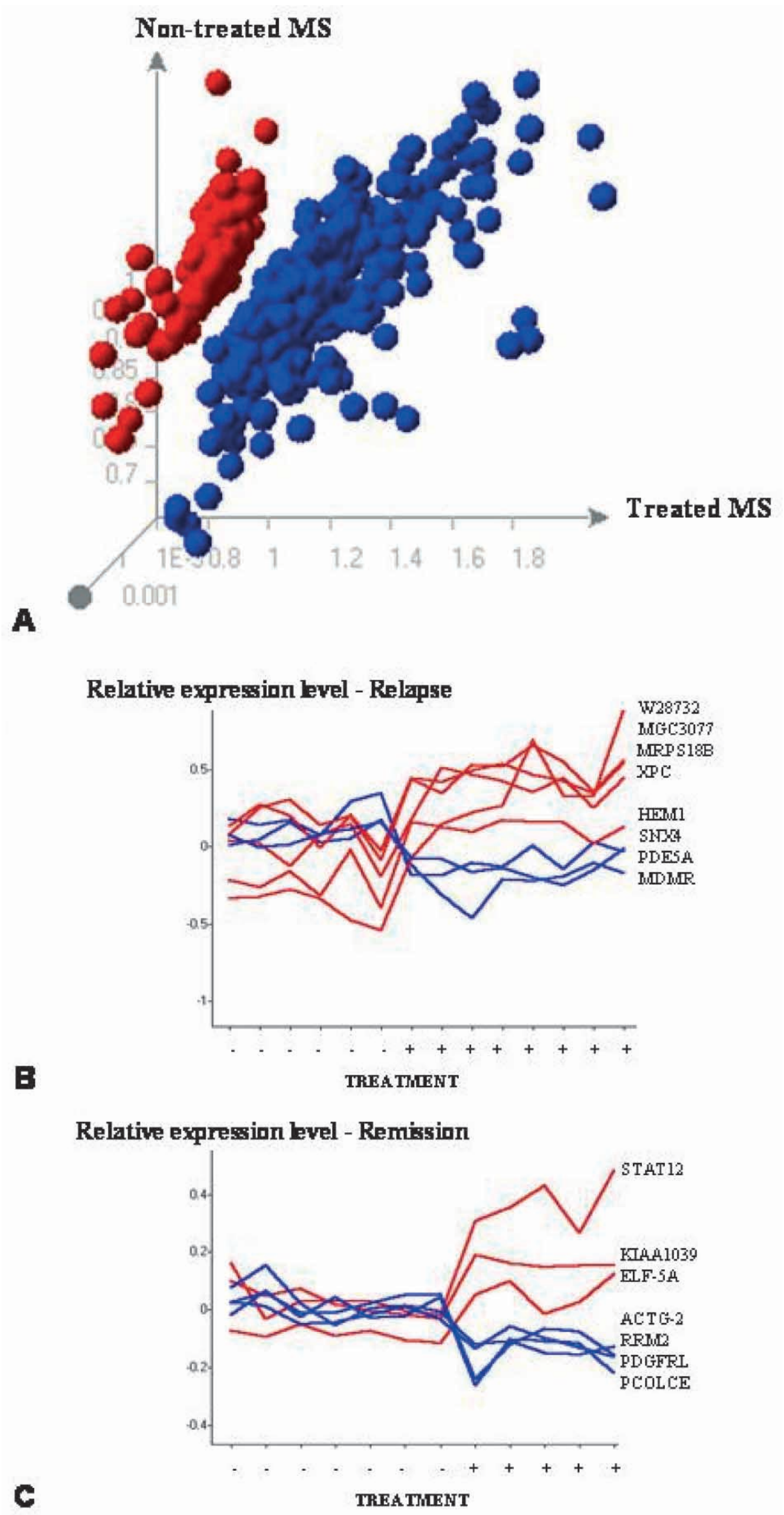

FIGURE 1 Immunomodulatory treatment effect evaluated by gene expression. (A) Three dimensional scatter plot of 535 significant genes ($p < 0.05$) demonstrating treatment effects in MS patients. $y$-axis denotes average fold change of each gene in non-treated patients, $x$-axis denotes average fold change of each gene in treated patients, both in comparison with healthy subjects, $z$-axis denotes $p$-value by Info. Red color represents over-expressed genes in untreated patients; blue color represents over-expressed genes in treated patients. (B) Profiles of abundant genes distinguishing treatment effects in MS patients during relapse. (C) Profiles of abundant genes distinguishing treatment effects in MS patients during remission.

TABLE I List of 30 cytokine-related gene transcripts in the MS specific signature

| Gene identifier | TNOM *p*-value | Info *p*-value | *t*-test *p*-value | Gene symbol | Gene name |
|---|---|---|---|---|---|
| X52560 | 4.82E-03 | 4.47E-03 | 2.97E-04 | CEBPB | CCAAT/enhancer binding protein, beta |
| L06797 | 1.38E-03 | 4.99E-04 | 1.35E-03 | CXCR4 | Chemokine, receptor 4 |
| AF046059 | 4.82E-03 | 4.02E-04 | 7.65E-04 | CREME9 | Cytokine receptor-like factor 3 |
| U56998 | 1.38E-03 | 4.67E-05 | 6.69E-05 | CNK | Cytokine-inducible kinase |
| D86964 | 4.82E-03 | 4.16E-03 | 1.47E-04 | DOCK2 | Dedicator of cyto-kinesis 2 |
| U43774 | 1.38E-03 | 1.96E-03 | 2.13E-04 | FCAR | Fc fragment of IgA, receptor |
| X54489 | 1.38E-03 | 5.88E-04 | 1.20E-03 | GRO1 | GRO1 oncogene |
| M36820 | 2.11E-06 | 1.80E-06 | 1.70E-07 | GRO2 | GRO2 oncogene |
| M36821 | 3.44E-04 | 4.67E-05 | 2.46E-06 | GRO3 | GRO3 oncogene |
| M94630 | 4.82E-03 | 1.61E-03 | 7.90E-04 | HNRPD | Heterogeneous nuclear ribonucleoprotein D |
| M27492 | 3.44E-04 | 7.05E-06 | 5.15E-06 | IL1R1 | Interleukin 1 receptor, type I |
| X04500 | 8.55E-11 | 8.55E-11 | 3.49E-12 | IL1B | Interleukin 1, beta |
| U32324 | 2.11E-06 | 4.08E-07 | 3.17E-08 | IL11RA | Interleukin 11 receptor, alpha |
| AF031167 | 1.38E-03 | 2.50E-03 | 2.15E-04 | IL15 | Interleukin 15 |
| M26062 | 4.82E-03 | 4.16E-03 | 9.97E-04 | IL2RB | Interleukin 2 receptor, beta |
| X04430 | 7.44E-05 | 1.29E-04 | 4.21E-06 | IL6 | Interleukin 6 (interferon, beta 2) |
| M17017 | 3.44E-04 | 5.23E-05 | 3.08E-06 | IL8 | Interleukin 8 |
| AB000734 | 1.37E-05 | 1.28E-06 | 2.07E-07 | SSI-1 | JAK binding protein |
| L36719 | 7.44E-05 | 7.05E-06 | 9.02E-07 | MAP2K3 | Mitogen-activated protein kinase kinase 3 |
| L41067 | 1.38E-03 | 2.21E-03 | 1.54E-04 | NFATC3 | Nuclear factor of activated T-cells |
| U02020 | 1.38E-03 | 1.96E-03 | 4.96E-05 | PBEF | Pre-B-cell colony-enhancing factor |
| AB014519 | 1.38E-03 | 1.35E-03 | 4.99E-04 | ROCK2 | Rho-associated, coiled-coil protein kinase 2 |
| M26683 | 4.82E-03 | 1.35E-03 | 1.25E-03 | SCYA2 | Small inducible cytokine A2 |
| J04130 | 7.44E-05 | 4.67E-05 | 5.86E-06 | SCYA4 | SCYA4 |
| U64197 | 8.55E-11 | 8.55E-11 | 6.81E-11 | SCYA20 | Small inducible cytokine subfamily A, 20 |
| U10117 | 1.37E-05 | 5.01E-06 | 1.91E-07 | SCYE1 | Small inducible cytokine subfamily E, 1 |
| AB004904 | 3.44E-04 | 1.90E-04 | 8.35E-04 | SSI-3 | STAT induced inhibitor 3 |
| D38122 | 4.82E-03 | 4.16E-03 | 8.10E-05 | TNFSF6 | Tumor necrosis factor superfamily, member 6 |
| S76792 | 1.37E-05 | 2.58E-05 | 4.27E-05 | TNFRSF4 | Tumor necrosis factor receptor superfamily, 4 |
| X02910 | 7.44E-05 | 1.19E-05 | 2.20E-07 | TNF | Tumor necrosis factor, 2 |

one of a family of 13 human CXC chemokines and is secreted by several types of cells in response to inflammatory stimuli. SCYA2 serves as chemotactic factor that attracts monocytes, binds to CCR2 and CCR4, and is implicated in the pathogenesis of autoimmune diseases like psoriasis, and rheumatoid arthritis. SCYA4 is a monokine with inflammatory and chemokinetic properties that binds to CCR5 and to CCR8 and was expressed both in the MS disease specific signature and in the immunomodulatory treatment signature, suggesting a combined effect of this gene transcript. SCYA20 is also a chemotactic factor for lymphocytes that binds to CCR6.

### T-cell Activation and Expansion

The second mechanism to operate is related to expansion of autoreactive T-cells, and involves several gene transcripts. The interleukin family (IL1b, IL6, IL8, IL15) and their receptors (IL1R1, IL11RA, IL2RB) have both a pro-inflammatory and anti-inflammatory activities, and are known to modulate the immune response and influence autoimmune activity. IL2RB plays a role in T-cell mediated immune response and is involved in receptor-mediated endocytosis and transduces the mitogenic signals of IL2. Its down-expression in the MS signature is in agreement with the findings reported by Suzuki *et al.* (1995), that demonstrated in IL2RB deficient mice that IL2RB is required to keep the activation program of T-cells under control and prevent autoimmunity. Additionally, the production of SCYA2 is regulated by IL2RB (Corrigall *et al.*, 2001). Another component of the interleukin family, IL15, is known to stimulate the proliferation of T-lymphocytes. Stimulation by IL15 requires interaction of IL15 with components of IL2 receptor, including IL2RB and possibly IL2RG. Another gene, NFATC3, also plays a role in the inducible expression of cytokine genes in response to antigenic stimulation of T-cells, especially in response to IL2 induction.

TABLE II List of seven cytokine-related gene transcripts in the MS treatment signature

| Gene identifier | TNOM *p*-value | Info *p*-value | *t*-test *p*-value | Gene symbol | Gene name |
|---|---|---|---|---|---|
| L22342 | 2.87E-03 | 4.47E-03 | 2.57E-03 | IFI41 | Interferon-induced protein 41 |
| J04130* | 1.26E-02 | 1.50E-02 | 6.30E-02 | SCYA4 | SCYA4 |
| D11086 | 4.43E-02 | 1.17E-02 | 3.40E-02 | IL2RG | Interleukin 2 receptor, gamma |
| X63717 | 4.43E-02 | 1.17E-02 | 3.95E-02 | TNFRSF6 | Tumor necrosis factor receptor superfamily, 6 |
| AI263885 | 4.43E-02 | 1.17E-02 | 1.34E-02 | WSX-1 | Class I cytokine receptor |
| L33404 | 4.43E-02 | 4.46E-02 | 7.02E-02 | KLK7 | Kallikrein 7 |
| U43774* | 4.43E-02 | 2.75E-02 | 7.51E-03 | FCAR | Fc fragment of IgA, receptor |

* Gene transcripts common to both MS disease related signature and MS immunomodulatory treatment signature.

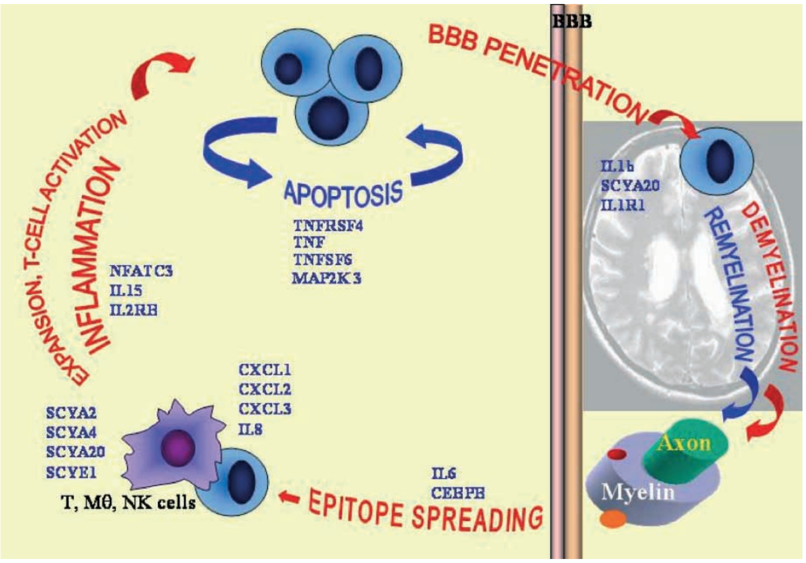

FIGURE 2 Principle scheme of the cytokine-related pathways involved in MS pathogenesis. BBB-blood brain barrier; NK-natural killer; MO-monocytes.

### Apoptosis

A breakdown in apoptosis related signaling mechanisms could result in the development of autoimmune disorders. Accumulating data indicate that impaired apoptosis plays a major role in the pathogenesis of MS. The group of TNF family related genes (TNF, TNFSF6, TNFRSF4) has multifunctional immune activities. TNFSF6 is a cytokine and an apoptotic factor that binds to FAS antigen and transduces the apoptotic signal into cells. It serves as a costimulatory molecule during T-cell activation, is involved in cytotoxic T-cell mediated apoptosis and in T-cells development (Embree-Ku *et al.*, 2002; Bolstad *et al.*, 2003; Linkermann *et al.*, 2003). Through FAS-antigen mediated apoptosis TNFSF6 may have a role in the induction of peripheral tolerance or induction of antigen stimulated suicide of mature T-cells. Its under-expression in the MS signature suggests impairment in immune tolerance and apoptosis that lead to persistent autoimmune activity. TNF was described as reducing the severity of prototypic Th1 diseases including EAE, and as inhibiting TCR signaling and promoting Th2 cytokine production (O'Shea *et al.*, 2002). It was recently reported that treatment with interferon beta in MS patients resulted in up-regulation of TNF inducing ligand (Wandinger *et al.*, 2003), thus it could be important to apoptotic associated pathways in MS.

### Demyelination

Another important mechanism that is disturbed in MS is related to remyelination and recovery. IL1β, previously reported to promote remyelination in a model of massive demyelination in mice, and IL1$\beta^{-/-}$ mice failed to remyelinate properly (Mason *et al.*, 2001). Its down expression within the MS signature suggests impaired remyelination that could be corrected by immunomodulatory treatment. Recently, it was suggested that IL-1RA and IL-1β are markers for MS severity and that a specific IL-1RA/IL-1β ratio was associated with worse prognosis of the disease (Schrijver *et al.*, 1999).

### Immune Regulation

CEBPB is an important transcriptional activator in the regulation of genes involved in immune and inflammatory responses. It specifically binds to an IL1 response element in the IL6 gene and might influence regulation of immune response both in the periphery and in the central nervous system. IL6 is an immunoregulatory cytokine. It was demonstrated that the failure of IL6-deficient mice to overcome regulatory T-cell-mediated suppression resulted in increased susceptibility to infection and resistance to autoimmunity (Pasare and Medzhitov, 2003). Similarly, in our study down expression of IL6 suggests impairment in immune regulation that might enhance the autoimmune process and epitope spreading in MS.

To summarize, we conclude that cytokine-associated genes are involved in different immune mechanisms whereby, autoreactive T-cells not normally deleted or destroyed can propagate and lead to active demyelination in MS. Future studies using gene microarray could be used to examine MS related mechanisms as well as the action of immunomodulatory treatments to optimize treatment responses and to better understand the disease process.

### *Acknowledgements*

The study was supported by the Rothberg Foundation and Mayer Research Grants.

### *References*

Achiron, A., Gurevich, M., Friedman, N., Kaminski, N. and Mandel, M. (2004) "Blood transcriptional signatures of multiple sclerosis: unique gene expression of disease activity", *Ann. Neurol.* **55**, 410–417.

Baranzini, S.E. and Hauser, S.L. (2002) "Large-scale gene-expression studies and the challenge of multiple sclerosis", *Genome Biol.* **3**, 1027.1–1027.5.

Bolstad, A.I., Eiken, H.G., Rosenlund, B., Alarcon-Riquelme, M.E. and Jonsson, R. (2003) "Increased salivary gland tissue expression of Fas ligand, cytotoxic T-lymphocyte-associated antigen 4, and programmed cell death 1 in primary Sjogren's syndrome", *Arthritis Rheum.* **48**, 174–185.

Corrigall, V.M., Arastu, M., Khan, S., *et al.* (2001) "Functional IL-2 receptor beta (CD122) and gamma (CD132) chains are expressed by fibroblast-like synoviocytes: activation by IL-2 stimulates monocyte chemoattractant protein-1 production", *J Immunol.* **166**, 4141–4147.

Dhib-Jalbut, S. (2002) "Mechanisms of action of interferons and glatiramer acetate in multiple sclerosis", *Neurology* **58**(8 suppl. 4), S3–S9.

Embree-Ku, M., Venturini, D. and Boekelheide, K. (2002) "Fas is involved in the p53-dependent apoptotic response to ionizing radiation in mouse testis", *Biol. Reprod.* **66**, 1456–1461.

Glabinski, A.R., Tuohy, V.K. and Ransohoff, R.M. (1998) "Expression of chemokines RANTES, MIP-1alpha and GRO-alpha correlates with inflammation in acute experimental autoimmune encephalomyelitis", *Neuroimmunomodulation* **5**, 166–171.

Kaminski, N. and Friedman, N. (2002) "Practical approaches to analyzing results of microarray experiments", *Am. J. Respir. Cell. Mol. Biol.* **27**, 125–132.

Kenealy, S.J., Pericak-Vance, M.A. and Haines, J.L. (2003) "The genetic epidemiology of multiple sclerosis", *J Neuroimmunol* **143**, 7–12.

Kolbert, C.P., Taylor, W.R., Krajnik, K.L. and O'Kane, D.J. (2003) "Gene expression microarrays", *Methods Mol. Med.* **85**, 239–255.

Li, C. and Wong, W.H. (2001) "Model-based analysis of oligonucleotide arrays: expression index computation and outlier detection", *Proc. Natl Acad. Sci. USA* **98**, 31–36.

Linkermann, A., Qian, J. and Janssen, O. (2003) "Slowly getting a clue on CD95 ligand biology", *Biochem. Pharmacol.* **66**, 1417–1426.

Martino, G., Furlan, R., Brambilla, E., *et al.* (2000) "Cytokines and immunity in multiple sclerosis: the dual signal hypothesis", *J. Neuroimmunol.* **109**, 3–9.

Mason, J.L., Suzuki, K., Chaplin, D.D. and Matsushima, G.K. (2001) "Interleukin-1beta promotes repair of the CNS", *J. Neurosci.* **21**, 7046–7052.

O'Shea, J.J., Ma, A. and Lipsky, P. (2002) "Cytokines and autoimmunity", *Nat. Rev. Immunol.* **2**, 37–45.

Ozenci, V., Kouwenhoven, M. and Link, H. (2002) "Cytokines in multiple sclerosis: methodological aspects and pathogenic implications", *Mult. Scler.* **8**, 396–404.

Pasare, C. and Medzhitov, R. (2003) "Toll pathway-dependent blockade of CD4+ CD25+ T cell-mediated suppression by dendritic cells", *Science* **299**, 1033–1036.

Prat, E. and Martin, R. (2002) "The immunopathogenesis of multiple sclerosis", *J. Rehabil. Res. Dev.* **39**, 187–199.

Schrijver, H.M., Crusius, J.B., Uitdehaag, B.M., *et al.* (1999) "Association of interleukin-1beta and interleukin-1 receptor antagonist genes with disease severity in MS", *Neurology* **52**, 595–599.

Suzuki, H., Kundig, T.M., Furlonger, C., *et al.* (1995) "Deregulated T cell activation and autoimmunity in mice lacking interleukin-2 receptor beta", *Science* **268**, 1472–1476.

Trapp, B.D. (2004) "Pathogenesis of multiple sclerosis: the eyes only see what the mind is prepared to comprehend", *Ann. Neurol.* **55**, 455–457.

Tsai, H.H., Frost, E., To, V., *et al.* (2002) "The chemokine receptor CXCR2 controls positioning of oligodendrocyte precursors in developing spinal cord by arresting their migration", *Cell* **110**, 373–383.

Wandinger, K.P., Lunemann, J.D., Wengert, O., *et al.* (2003) "TNF-related apoptosis inducing ligand (TRAIL) as a potential response marker for interferon-beta treatment in multiple sclerosis", *Lancet* **361**, 2036–2043.

Watson, A., Mazumder, A., Stewart, M. and Balasubramanian, S. (1998) "Technology for microarray analysis of gene expression", *Curr. Opin. Biotechnol.* **9**, 609–614.

Wolpe, S.D., Sherry, B., Juers, D., Davatelis, G., Yurt, R.W. and Cerami, A. (1989) "Identification and characterization of macrophage inflammatory protein 2", *Proc. Natl Acad. Sci.* **86**, 612–616.

